# Noninvasive detection of lung cancer by analysis of exhaled breath

**DOI:** 10.1186/1471-2407-9-348

**Published:** 2009-09-29

**Authors:** Amel Bajtarevic, Clemens Ager, Martin Pienz, Martin Klieber, Konrad Schwarz, Magdalena Ligor, Tomasz Ligor, Wojciech Filipiak, Hubert Denz, Michael Fiegl, Wolfgang Hilbe, Wolfgang Weiss, Peter Lukas, Herbert Jamnig, Martin Hackl, Alfred Haidenberger, Bogusław Buszewski, Wolfram Miekisch, Jochen Schubert, Anton Amann

**Affiliations:** 1 Department of Operative Medicine, Innsbruck Medical University, A-6020 Innsbruck, Austria; 2 Breath Research Unit of the Austrian Academy of Sciences, A-6850 Dornbirn, Austria; 3 Landeskrankenhaus Natters, A-6161 Natters, Austria; 4 Univ.-Klinik für Innere Medizin 5 (Hämatologie und Onkologie), Innsbruck Medical University, Anichstrasse 35, A-6020 Innsbruck, Austria; 5 Universitätsklinik für Strahlentherapie-Radioonkologie Innsbruck, Innsbruck Medical University, A-6020 Innsbruck, Austria; 6 Faculty of Chemistry, Nicolaus Copernicus University, PL-87100 Toruñ, Poland; 7 Institution of Radio-Oncology, LKH Vöcklabruck, A-4840 Vöcklabruck, Austria; 8 Department of Anaesthesiology and Intensive Care, University of Rostock, D-18057 Rostock, Germany

## Abstract

**Background:**

Lung cancer is one of the leading causes of death in Europe and the western world. At present, diagnosis of lung cancer very often happens late in the course of the disease since inexpensive, non-invasive and sufficiently sensitive and specific screening methods are not available. Even though the CT diagnostic methods are good, it must be assured that "screening benefit outweighs risk, across all individuals screened, not only those with lung cancer". An early non-invasive diagnosis of lung cancer would improve prognosis and enlarge treatment options. Analysis of exhaled breath would be an ideal diagnostic method, since it is non-invasive and totally painless.

**Methods:**

Exhaled breath and inhaled room air samples were analyzed using proton transfer reaction mass spectrometry (PTR-MS) and solid phase microextraction with subsequent gas chromatography mass spectrometry (SPME-GCMS). For the PTR-MS measurements, 220 lung cancer patients and 441 healthy volunteers were recruited. For the GCMS measurements, we collected samples from 65 lung cancer patients and 31 healthy volunteers. Lung cancer patients were in different disease stages and under treatment with different regimes. Mixed expiratory and indoor air samples were collected in Tedlar bags, and either analyzed directly by PTR-MS or transferred to glass vials and analyzed by gas chromatography mass spectrometry (GCMS). Only those measurements of compounds were considered, which showed at least a 15% higher concentration in exhaled breath than in indoor air. Compounds related to smoking behavior such as acetonitrile and benzene were not used to differentiate between lung cancer patients and healthy volunteers.

**Results:**

Isoprene, acetone and methanol are compounds appearing in everybody's exhaled breath. These three main compounds of exhaled breath show slightly lower concentrations in lung cancer patients as compared to healthy volunteers (p < 0.01 for isoprene and acetone, p = 0.011 for methanol; PTR-MS measurements). A comparison of the GCMS-results of 65 lung cancer patients with those of 31 healthy volunteers revealed differences in concentration for more than 50 compounds. Sensitivity for detection of lung cancer patients based on presence of (one of) 4 different compounds not arising in exhaled breath of healthy volunteers was 52% with a specificity of 100%. Using 15 (or 21) different compounds for distinction, sensitivity was 71% (80%) with a specificity of 100%. Potential marker compounds are alcohols, aldehydes, ketones and hydrocarbons.

**Conclusion:**

GCMS-SPME is a relatively insensitive method. Hence compounds not appearing in exhaled breath of healthy volunteers may be below the limit of detection (LOD). PTR-MS, on the other hand, does not need preconcentration and gives much more reliable *quantitative *results then GCMS-SPME. The shortcoming of PTR-MS is that it cannot identify compounds with certainty. Hence SPME-GCMS and PTR-MS complement each other, each method having its particular advantages and disadvantages. Exhaled breath analysis is promising to become a future non-invasive lung cancer screening method. In order to proceed towards this goal, precise identification of compounds observed in exhaled breath of lung cancer patients is necessary. Comparison with compounds released from lung cancer cell cultures, and additional information on exhaled breath composition in other cancer forms will be important.

## Background

Lung cancer is one of the leading causes of death in Europe and the western world. At present, diagnosis of cancer very often happens late in the course of the disease since available diagnostic methods are not sufficiently sensitive and specific.

There is strong evidence to suggest that particulate cancers can be detected by molecular analysis of exhaled air [1-9]. Breath analysis represents a new diagnostic technique that is without risk for the patient even if repeated frequently and can provide information beyond conventional analysis of blood and urine [9-16]. It may even be applied with patients at an intensive care unit [17,18] or during surgery [19]. Also real-time analysis of exhaled breath during an ergometer test or during sleep are possible [15,20-22].

Current interest is not only focused on breath of lung cancer patients but also on emission of volatile organic compounds (VOCs) from lung cancer tissue and lung cancer cell cultures [23-26]. In our own investigations we observed the release (or consumption) of various compounds for the cancer cell lines A549, CALU-1 [25], NCI-H2087 [26] and NCI-H1066 (Sponring A, Filipiak W, Mikoviny T, Ager C, Schubert J, Miekisch W, Amann A, Troppmair J: Release of volatile organic compounds from the lung cancer cell line NCI-H1666 *in vitro*, submitted). It would be most interesting to investigate jointly the composition of exhaled breath of a particular patient and *in vitro *release of compounds from cancer cells obtained from the same patient (during a tumor resection).

Different analytical techniques have been used for analysis of exhaled breath and headspace of cancer cell cultures. One of the most useful technique is gas chromatography and mass spectrometry (GCMS) [5,7,9,11,18,23,27-35]. This technique gives the most detailed analytical information. Other techniques, such as sensor arrays [36] and proton transfer reaction mass spectrometry (PTR-MS) [8] or selected ion flow tube mass spectrometry (SIFT-MS) [37-39] do not need any preconcentration step and can work in *on-line *mode, even in breath-to-breath resolution.

In the present manuscript we present results obtained with GCMS with preconcentration by solid phase microextraction (SPME), and results obtained with PTR-MS. Both techniques have their advantages and shortcomings. PTR-MS measurements do not provide as much information as GCMS, but the technique is more easy to handle. Therefore the number of patients or volunteers investigated by PTR-MS in our laboratory is much higher than the number of persons investigated by GCMS.

It has been stated by Phillips et al., that 3481 different compounds have been observed in exhaled breath. Some of these compounds arise in higher concentrations in inhaled air than in exhaled breath, having thus "negative alveolar gradient" [40]. A total of 1753 different compounds was described as having "positive alveolar gradient", i.e., arising in higher concentration in exhaled breath than in inhaled air. Michael Phillips et al. used adsorption of exhaled breath on adsorbents (solid phase extraction, SPE) with subsequent thermodesorption (TD) and GC-MS-analysis [5,7,27,41,42]. In their work, identification of the peaks has been done by spectral library match only without confirmation of retention time.

Among sample preparation methods, solid phase microextraction (SPME) is one of the most frequently used. SPME offers a simple and inexpensive alternative to preconcentration on sorbent tubes followed by thermal desorption (SPE). SPME was developed in the late 1980s by Arthur and Pawliszyn [43,44]. This technique was successfully used in determination of volatile compounds in different matrices in environmental, toxicological, and pharmacological analysis. SPME has been also applied to analysis of VOCs in human breath [45].

For the validated identification of potential marker compounds, we prepared calibration mixtures of the respective pure compounds in order to determine the retention time *and *mass spectra. A particular focus of the present investigation was the comparison of smokers vs. non-smokers within the group of lung cancer patients, and the *exclusion *of smoking-related compounds from comparisons between lung cancer patients and healthy controls. We recently performed a PTR-MS investigation comparing healthy smokers with healthy non-smokers and identified 7 different mass-to-charge ratios (in the range of 20 ≤ m/z ≤ 231) showing statistically significant differences between smokers and non-smokers [34].

SPME-GCMS identifies compounds, but is a relatively insensitive method. Hence compounds not appearing in healthy volunteers' exhaled breath may appear at lower concentrations below the limit of detection (LOD). In addition, SPME-GCMS is only semiquantitative due to *competitive absorption *on the SPME-fibre. PTR-MS, on the other hand, does not need preconcentration and gives much more reliable *quantitative *results. The shortcoming of PTR-MS is that it cannot identify compounds with certainty, and several compounds may overlap on a particular mass-to-charge ratio [46]. Hence SPME-GCMS and PTR-MS complement each other, each method having its particular advantages and disadvantages.

## Methods

### GCMS analysis

The GCMS analysis was performed on Agilent 5975 Inert XL MSD coupled with 7890 A gas chromatograph (Agilent, Waldbronn, Germany) with split-splitless injector. The temperature of injector was 290°C. The splitless time was 1 min, while split ratio was 1:50. Helium was used as a carrier gas at velocity 0.8 ml min^-1^. The MS analyses were carried out in a full scan mode, with scan range 35 - 200 amu. A scan rate of 3.46 scan/s was applied. Electron impact ionisation at energy 70 eV was used for every measurement. The ion source, quadrupole and transfer line temperatures were maintained at 230°C, 200°C and 200°C, respectively. The acquisition of chromatographic data was performed by means of Chemstation (Agilent) and mass spectrum library NIST 2005 (Gatesburg, USA) was applied to identification. The 25 m × 0.32 mm × 5 μm capillary column CP-Porabond-Q (Varian Inc., Middelburg, The Netherlands) was used. Oven temperature programme was as follows: initial 90°C held for 7 min, then ramped 7°C min-^1 ^to 140°C, held for 7 min then ramped 15°C min-^1 ^to 260°C and held for 10 min.

An automatic SPME holder with Carboxen/polydimethylsiloxane (CAR/PDMS) fiber of 75 μm thickness was purchased from Supelco (Bellefonte, PA, USA). The sorption and desorption of analytes have been performed automatically by means of autosampler MPS 2XL (Gerstel, Mülheim an der Ruhr, Germany). SPME conditions were as follow: extraction time of 10 min at a temperature of 37°C. Desorption of volatiles from the fiber was placed in hot GC injector at 290°C, for 1 min. Prior every SPME sorption, the fiber was preconditioned in small needle heater (Gerstel, Mülheim an der Ruhr, Germany) at temperature 290°C, for 5 min.

20 ml headspace vials, teflon coated rubber septa and crimp caps were purchased from Gerstel (Mülheim an der Ruhr, Germany). Helium and nitrogen of purity 6.0 (i.e., 99.9999%) were purchased from Linde (Vienna, Austria) and 3 Lit Tedlar bags from SKC (Eighty Four, PA, USA). Gas tight syringes were purchased from Hamilton (Bonaduz, Switzerland) and 1 l gas bulb from Supelco (Bellefonte CA, USA).

We defined signal to noise ratio of 3 as the limit of detection (LOD) and signal to noise ratios of 9 as the limit of quantification (LOQ).

### PTR-MS analysis

Volatile organic compounds with a volume ratio up to 1:10^10^(*0.1 mm*^3^* of the compound to 1 m*^3 ^*air*; 0.1 parts-per-billion; 0.1 ppb) can be measured. The molecules are ionized by proton transfer from H_3_O^+^-ions produced in the ion source of the instrument with subsequent measurement by a Quadrupol-Mass-Spectrometer [47-49]. The proton transfer reaction only takes place if the proton affinity of the analyte is higher than that of water, hence it is possible to detect most aldehydes, ketones, alcohols, acids, esters and many unsaturated aromatic as well as N or S substituted hydrocarbons. The count rate of the primary ions (H_3_O^+^) was calculated taking the count rate at m/z 21 and m/z 37 into consideration. For compounds with unknown kinetic reaction constant, the nominal constant *k *= 2 × 10^-9 ^cm^3 ^sec^-1 ^allows to determine a first estimate of the respective concentration (based on the measured count rate).

To calculate the concentrations for the mentioned compounds (acetone, benzene, isoprene, methanol) correctly, we performed calibration series (under consideration of the specific reaction rate constant *k*). The results of the used calibration as well as the used reaction constant are given in Table 1.

**Table 1:** Kinetic reaction constants and calibration results for different compounds according to PTR-MS measurements.

Compound	m/z	kinetic rate constant **(taken from reference **[55]**)**	calibration factor*^)^
benzene	79	1.97·10^-9 ^cm^3 ^sec^-1^	0.95

isoprene	69	2.0·10^-9 ^cm^3 ^sec^-1^	2.26

acetone	59	3.9·10^-9 ^cm^3 ^sec^-1^	1.21

methanol	33	2.7·10^-9 ^cm^3 ^sec^-1^	0.69

acetonitrile	42	4.5·10^-9 ^cm^3 ^sec^-1^	1.24

*^) ^The "calibration factor" is the factor used to correct concentrations which are initially determined using the kinetic rate constants as taken from reference [55]. The large "calibration factor" for isoprene is due to fragmentation of protonated isoprene (m/z 69) towards m/z 41.

A high-sensitivity proton transfer reaction mass spectrometer (hs-PTR-MS, 3 turbopumps; Ionicon Analytic GMBH, Innsbruck, Austria) with Teflon rings (instead of Viton rings) was used. The PTR-MS showed a count rate of the primary ions (H_3_O^+ ^ions) of ~1.5 × 10^7 ^counts per second. The settings of the PTR-MS result in a count rate of H_2_O^.^H_3_O^+ ^at around 7.5 × 10^4 ^and the percentage of parasitic precursor ions O_2_^+ ^<1%, NO^+^<0.5% and NH_4_^+ ^< 8% (the given values refer to dried, filtered room air, so-called *zero-air*). For additional details see references [46,50].

### Reagents and standards

Acetaldehyde, 2-butanone and 2-pentanone were bought from Acros Organics (Geel, Belgium) 2-methyl-1-butene from Chemsampco (Dallas, TX, USA), and rest of compounds were purchased from Sigma-Aldrich (Steinheim, Germany).

### Preparation of gaseous standards

Calibration gases were prepared mostly by evaporation of liquid compounds (except C4-C6 alkanes, 2-butene and dimethyl ether) in a glass gas bulb. Before using, all bulbs had been cleaned with methanol, dried in oven at 120°C for at least 20 h, and then purged with ultra-clean nitrogen for at least 10 min. Afterwards, bulbs were evacuated by means of a vacuum pump for 10 min. Calibration vapour mixture was obtained by injection of 1-3 μl of each compound through membrane, using a GC syringe into a glass bulb. After evaporation, appropriate amount of vapour mixture was removed using gas tight syringe and introduced into Tedlar^® ^bags (SKC 232 Series, Eighty Four, PA, USA) with 0.5, 1 or 1.5 L of nitrogen.

### Human subjects: GCMS analysis

A cohort of 65 patients (28 smokers, 31 exsmokers and 6 nonsmokers) suffering from lung cancer at different stages and in different treatment regimes (median age 63.0 years and an age range of 37 - 84 years) was recruited. All individuals gave informed consent to participation in the study. The patients completed a questionnaire describing their current smoking status (active smokers, non-smokers) and the time elapsed since their last smoke. The classification as smoker/non-smoker/ex-smoker is based on the self-declaration of the patients. The amount of smoking (in pack years) was determined.

The lung cancer patients were compared with 31 healthy volunteers (7 smokers, 2 exsmokers and 22 nonsmokers, median age 38.0 years and an age range of 21 - 87 years), who also gave informed consent to participation in the study and declared their smoking habits.

All patients and volunteers consumed food not later than one hour before breath sampling. No special dietary regimes were applied. The samples were collected at different daytime independent of the time of meals and were processed within 6 hours at most. The study was approved by the local ethics committee of Innsbruck Medical University.

### Human subjects: PTR-MS analysis

A cohort of 220 lung cancer patients (68 smokers, 129 exsmokers and 23 nonsmokers) at different stages and in different treatment regimes (median age 64.4 years and an age range of 39 - 83 years) and 441 healthy volunteers (84 smokers, 86 exsmokers and 271 nonsmokers, median age 54 years and an age range of 17 - 91 years) was recruited. Recruitment and protocol is identical to the patients and volunteers measured by GCMS.

### Sampling of exhaled breath

Samples of mixed breath gas were collected in Tedlar bags (SKC Inc, Eighty Four, PA) with parallel collection of ambient air (also in Tedlar bags). Breath gas samples were obtained after a ~5 minutes sitting of a volunteer. Each subject provided 1 or 2 breath samples by use of a straw. All samples were processed within 3-6 hours. We collected mixed alveolar breath (instead of alveolar breath) in order to find also compounds directly released from the lungs.

Before collection of breath, all bags were thoroughly cleaned to remove any residual contaminants by flushing with nitrogen gas (purity of 99.9999%), and then finally filled with nitrogen and heated at 85°C for more than 8 hours with a complete evacuation at the end.

All compounds detected in breath were compared to the ambient air and only compounds with concentrations *at least 15% higher *than in ambient air concentrations were reported. 18 ml of gas sample has been transferred to 20 ml volume evacuated glass vials, and equilibrated with nitrogen gas.

Here we determined VOCs in exhaled breath of lung cancer patients. We restrict ourselves to compounds which show at least 15% higher concentrations in exhaled breath as compared to inhaled air. In particular, we exclude compounds which show lower concentration in exhaled breath than in inhaled air. Our experience showed that different rooms show quite different indoor air composition, which is particularly pronounced in clinical environments. The threshold of 15% was arbitrarily chosen.

### Smokers evaluation (GCMS measurements)

**Figure 1:**
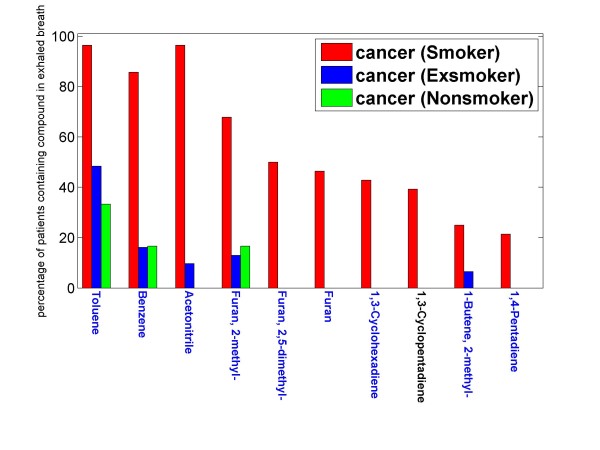
**Compounds observed in exhaled breath of *cancer patients *which are related to *smoking behavior *(GCMS)**. Only those measurements have been considered, which show at least 15% higher concentration in exhaled air than in indoor air. The relative proportions for observations with respect to smoker patients (red), ex-smoker patients (blue) and non-smoker patients (green) is shown. Nine compounds (blue) were identified by retention time *and *by spectral library identification. One compound, namely 1,3-cyclopentadiene was not commercially available and therefore only identified by spectral library match. Acetonitrile and toluene arise in almost every smokers' exhaled breath. p-Values computed according to the method of Agresti and Caffo [51] are smaller than 0.0001 with the exception of 2-methyl-1-butene and 1,4-pentadiene, where p < 0.05.

For each compound found, we determined the proportion of lung cancer patients in whose exhaled breath the compound appears, separately for smokers, exsmokers and non-smokers, see Figure 1. Putting smokers into one class and combining exsmokers and non-smokers into the other class, the respective proportions, namely proportion_smoker _and proportion_exsmoker *and *non-smoker _were computed for appearance of each compound. The p-value for the null hypothesis **"proportion_smoker _= proportion_exsmoker *and *non-smoker_" **was computed according to the method proposed by Agresti and Caffo [51]. Statistical results are considered to be significant if *p <*0.05.

### Group comparisons (PTR-MS measurements)

The non-parametric Kruskal-Wallis test (equivalent to the Wilcoxon rank sum test) was used for all comparisons of concentrations in two groups. For all comparisons, the null-hypothesis was that the concentrations of compared groups are equally distributed. Statistical results are considered to be significant if *p <*0.01. We chose this limit (instead of p < 0.05) because of the large number of subjects in the cohort investigated by PTR-MS (220 lung cancer patients, 441 healthy controls).

### Compounds excluded for distinction of lung cancer patients from healthy volunteers

Certain compounds may arise in relatively high concentrations in indoor air. Typical examples are isopropanol or limonene (from cleaning agents) or p-xylene (in hospital indoor air). Other compounds like cineole, menthol, p-cymene or ethanol may be contained in toothpaste, candies or foodstuff. Phenol and N,N-dimethyl acetamide are released from Tedlar bags and carbon disulfide is frequently released by GCMS septa. Halogenated compounds like trichloroethylene, acetylbromide or 1,1-difluoroethane have to be treated with care. N,N-dimethyl formamide may also have a hospital-related background. Other compounds like acetonitrile are typical for smoking (see Results Section). These mentioned compounds were excluded when trying to distinguish lung cancer patients from healthy volunteers with respect to VOC concentration patterns in exhaled breath.

## Results

### Patient characterization

The demographic data of patients, volunteers and the histologic subtypes of lung cancer are presented in Table 2, 3, 4, and 5.

**Table 2:** Demographic data related to current smoking status for control and cancer patients measured by PTR-MS*).

		Smokers		Non-smokers		Ex-smokers		Total	
		
		n	Age Median (range)	n	Age Median (range)	n	Age Median (range)	n	Age Median (range)
Control	Female	56 (55)	43 (18-81)	176 (171)	57.5 (17-91)	39 (37)	55 (25-85)	271 (263)	53.83 (17-91)
	
	Male	28 (26)	46 (23-77)	95 (91)	53 (24.5-86)	47 (47)	64 (33-88)	170 (164)	56.25 (23-88)
	
	Total	84 (81)	44 (18-81)	271 (262)	57 (17-91)	86 (84)	61.25 (25-88)	441 (427)	54 (17-91)

CA- Patients	Female	23 (23)	55 (46-77)	18 (17)	71 (39-82.5)	39 (39)	64.5 (44-83)	80 (79)	63 (39-83)

	Male	45 (43)	60 (40-77)	5 (5)	61 (51-70)	90 (88)	66.3 (46.4-81)	140 (136)	65 (40-81)

	Total	68 (66)	58 (40-77)	23 (22)	67 (39-82.5)	129 (127)	66 (44-83)	220 (215)	64.4 (39-83)

*) Some measurements for PTR-MS were excluded (because of device variations), in brackets the number of subjects were at least 1 measurement was valid is given.

**Table 3:** Demographic data related to current smoking status for control and cancer patients measured by GCMS.

		Smokers		Non-smokers		Ex-smokers		Total	
		
		n	Age Median (range)	n	Age Median (range)	n	Age Median (range)	n	Age Median (range)
Control	Female	4	52.5 (21 - 65)	10	51.0 (23 - 87)	2	57.5 (30 - 85)	16	51.0 (21 - 87)
	
	Male	3	26.0 (25 - 28)	12	35.5 (27 - 68)	0		15	33.0 (25 - 68)
	
	Total	7	28.0 (21 - 65)	22	41.5 (23 - 87)	2	57.5 (30 - 85)	31	38.0 (21 - 87)

CA-patients	Female	10	60.5 (37 - 84)	5	59.0 (48 - 82)	9	58.0 (48 - 72)	24	58.5 (37 - 84)
	
	Male	18	59.0 (45 - 78)	1	79.0 (79 - 79)	22	65.0 (51 - 79)	41	64.0 (45 - 79)
	
	Total	28	59.0 (37 - 84)	6	66.5 (48 - 82)	31	65.0 (48 - 79)	65	63.0 (37 - 84)

**Table 4:** Histology of lung cancers (for patients whose exhaled breath was investigated by GCMS)

Small cell		15
Non-small cell	adenocarcinoma 25	

	epidermoid carcinoma 17	

	large cell carcinoma 1	

	mixed epidermoid-large cell carcinoma 4	47

Mesothelioma		1

Carcinoid		2

**Table 5:** Histology of lung cancers (for patients whose exhaled breath was investigated by PTR-MS)

Small cell		39
Non-small cell	adenocarcinoma 98	

	epidermoid carcinoma 64	

	large cell carcinoma 7	169

Mesothelioma		2

Carcinoid		5

Others		5

### Concentrations of volatile organic compounds as determined by PTR-MS

The distribution of concentrations for benzene, isoprene, acetone and methanol was determined by PTR-MS. The substances isoprene, acetone and methanol are the main components of exhaled breath.

**Figure 2:**
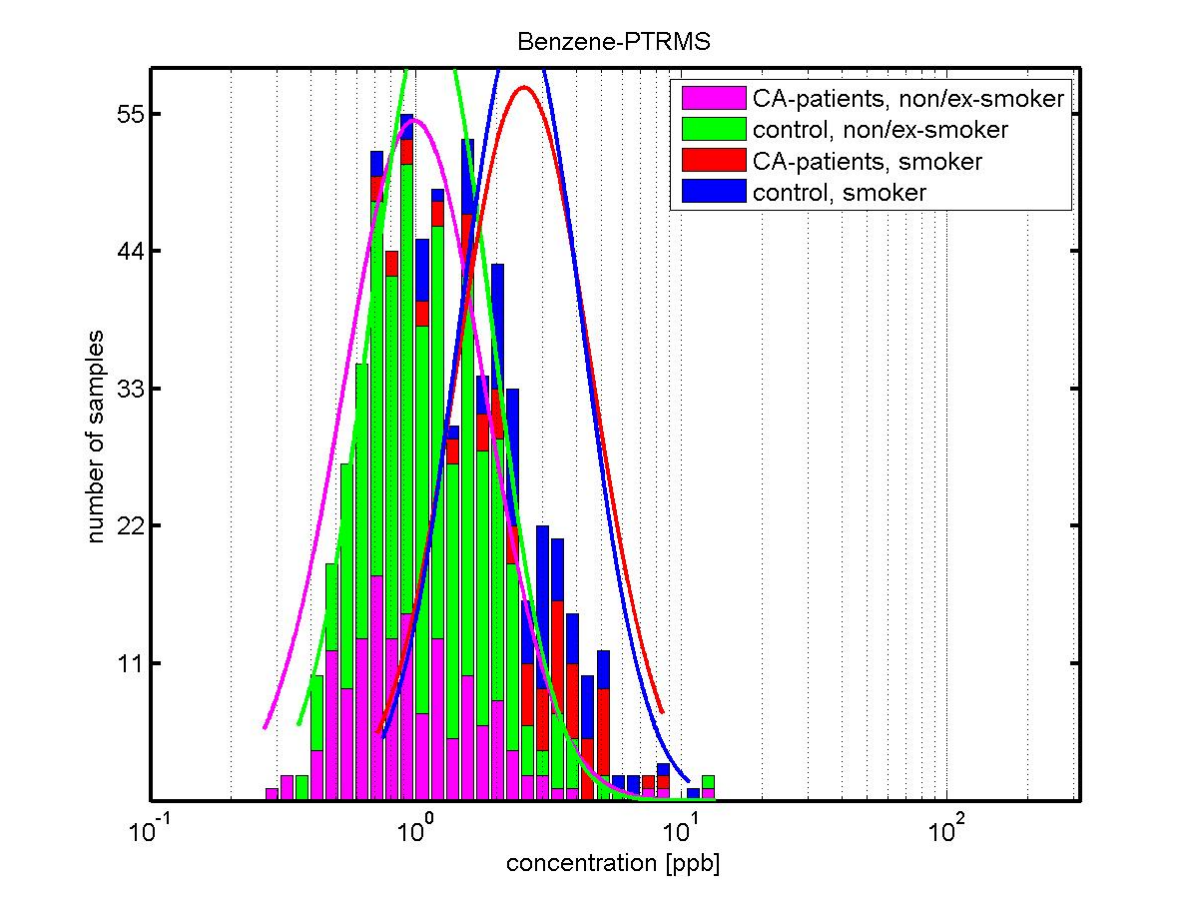
**Concentration distributions of benzene (determined by PTR-MS) in exhaled breath of lung carcinoma patients (smokers: red; non/ex-smoker: magenta) and healthy volunteers (smokers: blue; non/ex-smokers: green)**. The concentration is shown in logarithmic scaling. Benzene concentration is increased in the subgroups of smokers and therefore a marker for smoking behavior. The median values for benzene in human breath have been determined as follows: *CA-patients-non/ex-smoker *0.9 ppb, *control-non/ex smokers *1.1 ppb (significant difference according to Kruskal-Wallis test: p-value 0.002); *CA-patients-smoker *2.9 ppb, *control-smokers *2.4 ppb (no statistical significant difference according to Kruskal-Wallis test: p-value 0.469). The difference in smoking behaviour (between smoker and non/ex-smoker) is statistically significant for the control group (p-value < 1*10^-5^) as well as for the CA-patients (p-value < 1*10^-5^).

The results for *benzene *are shown in Figure 2. The median concentrations of benzene are increased in smokers (*CA-smoker *2.9 ppb; *control-smoker *2.4 ppb) as compared to non-smokers and exsmokers (*CA-nonsmoker/exsmoker *0.9 ppb, *control-nonsmoker/exsmoker *1.1 ppb). We observe a small difference between lung cancer patients and controls: statistically significant for the non/ex-smoker subgroups (p = 0.002), but not for the smoker-subgroup (p = 0.469).

**Figure 3:**
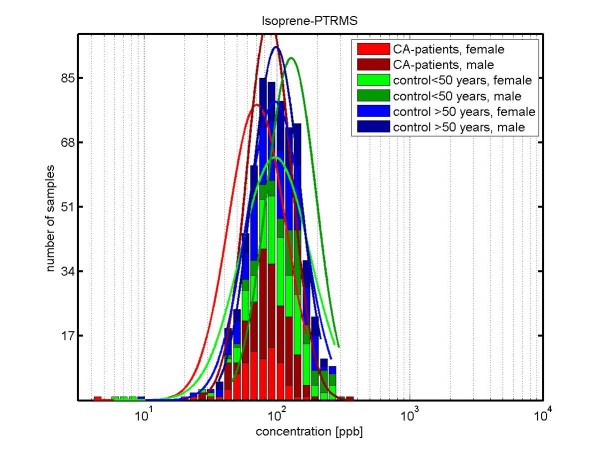
**Concentration distributions of isoprene (determined by PTR-MS) in exhaled breath of lung carcinoma patients (*CA-patients-female*: red; *CA-patients-male*: dark red) and healthy volunteers (*control < 50 years, female*: green; *control < 50 years, male*: dark green; *control > 50 years, female*: blue; *control > 50 years, male*: dark blue)**. The concentration is shown in logarithmic scaling. The median concentration of isoprene in exhaled breath of cancer patients is 81.5 ppb, whereas in healthy controls it is 105.2 ppb. For females, the median concentration for isoprene in breath is: for CA-patients 72.9 ppb; for control < 50 years 103.9 ppb and for controls > 50 years 98.1 ppb. For males, the median concentration for isoprene in breath is: for CA-patients 87.1 ppb; for control < 50 years 133.7 ppb and for controls > 50 years 100.0 ppb. Separated for gender, the difference for CA-patients and control, older 50 years was statistically significant for females: p-value < 1.0*10^-5^, but not for males: p-value = 0.022.

**Figure 4:**
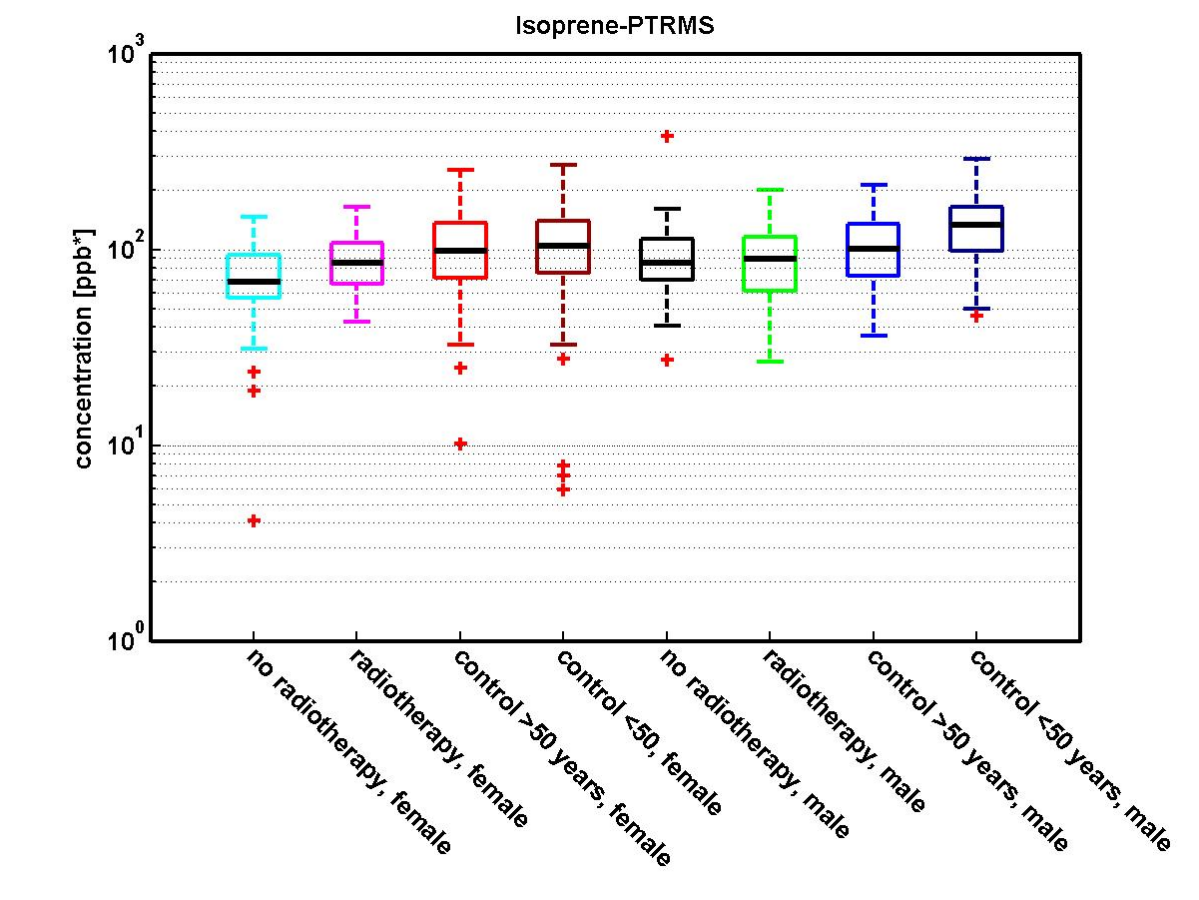
**Concentration distributions of isoprene (determined by PTR-MS) in exhaled breath of lung carcinoma patients treated with radiotherapy (*CA-patients-radiotherapy-female (n = 22)*: magenta; *CA-patients-radiotherapy-male (n = 36)*: green), carcinoma patients not treated with radiotherapy (*CA-patients-no radiotherapy-female (n = 50)*: cyan; *CA-patients-no radiotherapy-male (n = 88)*: black) and healthy volunteers (*control < 50 years, female (n = 121)*: dark red; *control < 50 years, male (n = 70)*: dark blue; *control > 50 years, female (n = 142)*: red; *control > 50 years, male (n = 94)*: blue)**. 19 CA-patients (7 female, 12 male) were excluded from consideration, because they did not clearly fit into one of the two groups (with/without radiotherapy). The concentration is shown in logarithmic scaling. For females, the median concentration for isoprene in breath is: for CA-patients-radiotherapy 85.1 ppb; for CA-patients-no radiotherapy 68.1 ppb; for control < 50 years 103.9 ppb and for controls > 50 years 98.1 ppb. For males, the median concentration for isoprene in breath is: for CA-patients-radiotherapy 89.9 ppb; for CA-patients-no radiotherapy 85.4 ppb; for control < 50 years 133.7 ppb and for controls > 50 years 100.0 ppb. A discussion of age- and gender effects in healthy volunteers is given in reference [33].

The results for *isoprene *are shown in Figures 3 and 4. The median concentrations of isoprene are lower in lung cancer patients (81.5 ppb) than in healthy controls (105.2 ppb), with a p-value *p *< 0.01. Since isoprene shows age and gender effects [33], we differentiated also with respect to gender, see Table 6: For *male persons *we observed median concentrations of 133.7 ppb in controls which are younger than 50 years, 100.0 ppb in controls which are older than 50 years, and 87.1 ppb in lung cancer patients. In *female persons *we observed median concentrations of 103.9 ppb in controls which are younger than 50 years, 98.1 ppb in controls which are older than 50 years, and 72.9 ppb in lung cancer patients. Additional information on lung cancer patients with and without radiotherapy are given in Figure 4: Lung cancer patients undergoing a radiotherapy show a slightly higher concentration of isoprene than lung cancer patients without radiotherapy.

**Table 6:** Statistical results related to the concentration of *isoprene *in exhaled breath (PTR-MS), whose concentrations show gender- and age-specific behavior [33].

compared populations	restricted to population	p-value*^)^ H0: equal mean values for log-transformed concentrations; H1: not equal mean values	median concentration of isoprene for population (A)	median concentration of isoprene for population (B)
Female (population A) vs. Male (population B)	Control, younger 50 years	0.0005	103.9 ppb	133.7 ppb
	
	Control, older 50 years	0.940, not significant	98.1 ppb	100.0 ppb
	
	CA-patients	0.0008	72.9 ppb	87.1 ppb

Control, younger 50 years (A) vs. control, older 50 years (B)	Female	0.564, not significant	103.9 ppb	98.1 ppb
	
	Male	0.0002	133.7 ppb	100.0 ppb

CA-patients (A) vs. control, older 50 years (B)	Female	1.0*10^-5^	72.9 ppb	98.1 ppb
	
	Male	0.022, not significant	87.1 ppb	100.0 ppb

*) according Kruskal-Wallis test, alpha-level = 0.01

**Figure 5:**
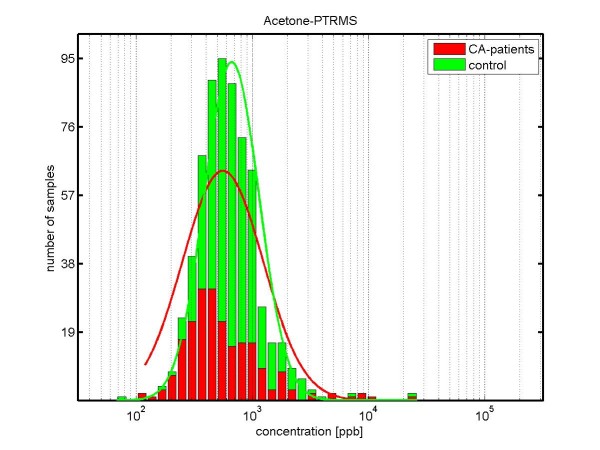
**Concentration distributions of acetone (determined by PTR-MS) in exhaled breath of lung carcinoma patients (red) and healthy volunteers (green)**. The concentration is shown in logarithmic scaling. The median concentration of acetone in exhaled breath of cancer patients is 458.7 ppb, whereas in healthy controls it is 627.5 ppb. (significantly different according Kruskal-Wallis test; p-value = 0.001).

The results for *acetone *are shown in Figure 5: The median concentrations of acetone are lower in lung cancer patients (458.7 ppb) than in healthy controls (627.5 ppb), with a p-value *p *< 0.01.

**Figure 6:**
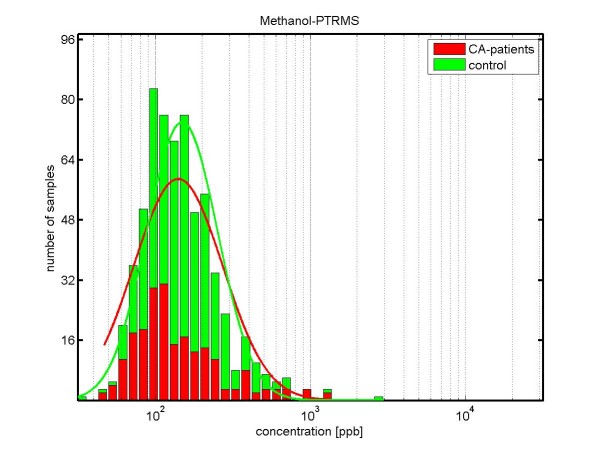
**Concentration distributions of methanol (determined by PTR-MS) in exhaled breath of lung carcinoma patients (red) and healthy volunteers (green)**. The concentration is shown in logarithmic scaling. The median concentration of methanol in exhaled breath of cancer patients is 118.5 ppb, whereas in healthy controls it is 142.0 ppb. (not significantly different according Kruskal-Wallis test; p-value = 0.011).

The results for *methanol *are shown in Figure 6: The median concentrations of methanol are lower in lung cancer patients (118.5 ppb) than in healthy controls (142.0 ppb), with a p-value *p *= 0.011.

### Volatile organic compounds in lung cancer patients observed by GCMS

We observed altogether 103 volatile organic compounds (VOCs) in exhaled breath of lung cancer patients. We do not claim that all of these compounds are endogenously produced. These compounds were identified by spectral library match. For 84 of these 103 compounds we confirmed identification by comparison of retention times with native standards.

Limit of detection (LOD) with our SPME-GCMS method was determined for 43 different compounds [35]. The lowest LOD was 0.7 ppb (for isoprene), the highest LOD was 17.2 ppb (for 3-Butyn-2-ol). The median LOD of 43 compounds was 2.4 ppb.

The appearance of the following compounds is influenced by smoking habits: toluene, benzene, acetonitrile, 2-methyl furan, 2,5-dimethyl furan, furan, 1,3-cyclohexadiene, 1,3-cyclopentadiene, 2-methyl-1-butene, 1,4-pentadiene. p-Values computed according to the method of Agresti and Caffo [51] are smaller than 0.0001 with the exception of 2-methyl-1-butene and 1,4-pentadiene, where p < 0.05. The proportion of lung cancer patients showing these compounds in their exhaled breath is shown in Figure 1 for smokers, ex-smokers and non-smokers. Apart from 1,3-cyclopentadiene (which is not commercially available), all these smoking-related compounds were not only identified by spectral library match, but also by comparison of GCMS retention time with that of standards based on the respective pure compound.

All the compounds related to smoking behavior were excluded from the list of compounds which are thought to be specific for lung cancer.

### VOCs specific for lung cancer patients (PTR-MS)

For isoprene, acetone and methanol we observe lower concentrations in exhaled breath of lung cancer patients as compared to healthy volunteers (see Discussion Section).

### VOCs specific for lung cancer patients (GCMS)

A comparison of the results of our cohort of 65 lung cancer patients with those of 31 healthy volunteers revealed differences in the concentration patterns. Sensitivity for detection of lung cancer patients based on 4 different compounds (or 15 different compounds or 21 different compounds) not arising in exhaled breath of healthy volunteers was 52% (71% and 80%). Compounds specific for smoking were *not *included in these sets. The three used sets of compounds were increasing, i.e.,

The smallest set-A of 4 compounds consisted of 2-butanone, benzaldehyde, 2,3-butanedione, 1-propanol. The other sets of compounds are listed in Table 7. All the 21 compounds used here for detection of lung cancer patients were not observed in healthy controls at concentrations at least 15% higher in exhaled breath than in indoor air (as detected by SPME with its relatively high LOD). Hence specificity is always 100% for all three sets of compounds.

**Table 7:** Potential marker compounds for lung cancer (GCMS), set-A consisting of 4 different compounds, set-B of 15 compounds, and set-C of 21 compounds.

	compound name	CAS-number	checked for retention time
**compounds for set A**	2-Butanone	78-93-3	1

	Benzaldehyde	100-52-7	1

	2,3-Butanedione	431-03-8	0

	1-Propanol	71-23-8	1

**add. compounds for set B**	2-Butanone, 3-hydroxy-	513-86-0	1

	3-Butyn-2-ol	2028-63-9	1

	Butane, 2-methyl-	78-78-4	1

	2-Butene, 2-methyl-	513-35-9	1

	Acetophenone	98-86-2	1

	1-Cyclopentene	142-29-0	1

	Methyl propyl sulfide	3877-15-4	1

	Urea, tetramethyl-	632-22-4	1

	n-Pentanal	110-62-3	1

	1,3-Cyclopentadiene, 1-methyl-	96-39-9	0

	2-Butanol, 2,3-dimethyl-	594-60-5	1

**add. compounds for set C**	Isoquinoline, 1,2,3,4-tetrahydro-	91-21-4	0

	Undecane, 3,7-dimethyl-	17301-29-0	0

	Benzene, cyclobutyl-	4392-30-7	0

	Butyl acetate	123-86-4	1

	Ethylenimine	151-56-4	0

	n-Undecane	1120-21-4	0

**Figure 7:**
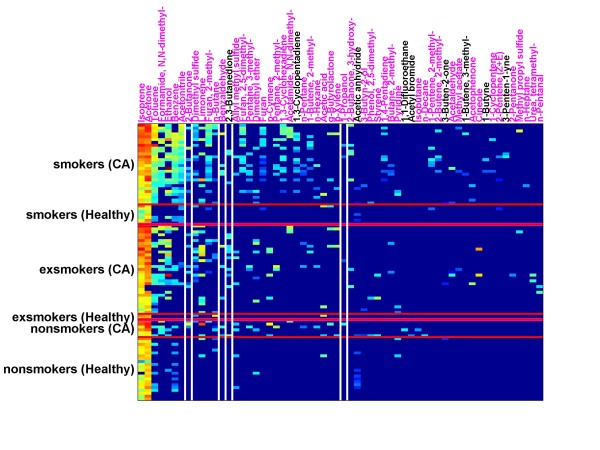
**This figure shows the distribution of appearance in exhaled breath of cancer patients (smokers, exsmokers and non-smokers) for 60 compounds out of altogether 103 compounds (GCMS)**. Isopropanol is not appearing in this figure, because it usually shows higher concentrations in indoor air than in exhaled air. Carbondisulfide is not appearing in this figure, because it may be released from septa used for SPME investigations. Names of those compounds which have been identified not only by spectral library match but *in addition by comparison with retention time of the pure respective compound*, are shown in magenta color. Names of compounds only identified by spectral library match are shown in black color. The columns of four suggested marker compounds (set-A) are shown between white vertical lines. These compounds appear in cancer patients (smokers, ex-smokers and non-smokers), but do not appear in exhaled breath of healthy volunteers with concentrations above the limit of detection (LOD).

Figure 7 illustrates the concentration patterns of selected compounds (including the above set-A of 4 compounds) for lung cancer smokers, lung cancer ex-smokers and lung cancer non-smokers as compared with healthy smokers, healthy ex-smokers and healthy non-smokers. Some compounds like isoprene and acetone arise in everybody's exhaled breath. Some compounds like acetonitrile are typical for smoking behavior. Some compounds were observed only in lung cancer patients (e.g., the sets A-C of compounds given in Table 7).

## Discussion

The following points characterize our investigation and are discussed here:

(A) *No differences *of concentrations (concentration_expiratory _- concentration_inspiratory_) between exhaled breath and indoor air were considered. Only expiratory concentrations (concentration_expiratory_) were considered. All our results refer to measurements which showed at least 15% higher concentrations in exhaled breath than in indoor air. If indoor air concentrations of some compound was higher than the concentration in exhaled air, the respective compound was not considered for the particular patient/volunteer in question.

(B) We *excluded *certain compounds from use for the distinction between lung cancer patients and healthy volunteers, because these compounds may be related to artefacts. These compounds may be observed due to indoor air contamination, cleaning agents, toothpaste, candies or foodstuff, compounds being released from tubings, Tedlar bags or GCMS septa etc, see Table 8.

**Table 8:** List of compounds which were *excluded *from consideration for the differentiation of lung cancer patients from healthy controls

1,1-difluoroethane	used as a refrigerant, hence an exogenous origin is possible
2-propanol	indoor air component in hospital rooms

2-propanol, 1,1,1-trichloro-2-methyl-	exogenous origin?

acetamide, N,N-dimethyl-	is released by Tedlar bags

acetic anhydride and acetyl bromide	unclear origin

benzene, ethyl-	potentially interesting compound, but one of the volatile BTEX-compounds (= benzene, toluene, ethyl-benzene, xylene) appearing in gasoline

carbon disulfide	is released by GCMS septa

cineole	used in flavorings, fragrances, and cosmetics

diethyl ether	suspected to be an indoor air component in hospital rooms

ethanol	could be of exogenous origin

ethylene, tetrachloro-	used in dry cleaning, hence an exogenous origin is possible

formamide, N,N -dimethyl-	suspected to be an indoor air component in hospital rooms

isobutane	exogenous origin? (propellant)

limonene	exogenous origin? (is used in food manufacturing, cosmetics and cleansing agents)

p-cymene	is contained in essential oils (e.g., in cumin and thyme)

m-cymene	misidentification possible (mix-up with natural isomer p-cymene)

menthol mix of isomers	might be contained in candies, toothpaste or foodstuff

methyl acetate	is observed in healthy volunteers in low concentration (ca. 1 ppb), and increases with increased cardiac output

n-hexane	there is an ubiquitous pollution with n-hexane in the environment

n-pentane	marker for oxidative stress

p-xylene	indoor air component in hospital rooms

pentane, 2-methyl- and pentane, 3-methyl	potentially interesting compound, but might be released by GCMS septa

styrene	styrene is sometimes added to the BTEX-compounds (see ethyl-benzene above), making it BTEXS

trichloroethylene	TCE; groundwater contamination by TCE is an important environmental concern, hence an exogenous origin is possible

(C) We were interested in compounds related to smoking behavior, but did *not *use them to distinguish between lung cancer patients and healthy volunteers.

(D) We determined retention times of as many compounds as possible based on standards prepared from the respective pure compound. In total, we determined retention times of ~220 compounds with respect to our SPME-GCMS measurement protocol. For the present study with 103 observed compounds altogether 84 compounds were confirmed by retention time. We clearly indicate for each compound if it was identified by spectral library match only, or additionally by determination of retention time.

(E) We strictly observed the policy that one GCMS-peak corresponds to one compound.

(F) We used Tedlar bags (polyvinylfluoride), glass vials and Teflon tubings. Our experience is that these materials have very limited release of volatile compounds (phenol and N,N-dimethyl acetamide for Tedlar bags). Other tubings than Teflon may release plasticizers. Phillips et al. [6] mention among marker compounds plasticizers such as "2,2,4-Trimethyl-1,3-pentanediol diisobutyrate", "Pentanoic acid, 2,2,4-trimethyl-3- carboxyisopropyl, isobutyl ester" and "Propanoic acid, 2-methyl-, 1-(1,1- dimethylethyl)-2-methyl- 1,3-propanediyl ester". It might be that these compounds are not of biological origin, but have their origin in the release of these compounds from some tubing system.

(G) All our GCMS-experiments described here were done using SPME. This is not as sensitive as solid phase extraction with subsequent thermodesorption. If measurements are done with higher sensitivity, certain compounds may be present in exhaled breath of healthy volunteers, even if we do not detect them by SPME.

The approach (A) for us is physiologically more informative than taking *differences *in concentrations whenever a VOC behaves like carbon dioxide: the concentration of CO_2 _in exhaled air (about 4%) is, within the normal inspiratory range, independent of the CO_2 _concentration in inhaled air (0.03% to 2% in indoor air) [52]. Only the exhaled concentration of CO_2 _(and not the difference of concentrations in exhaled breath and indoor air) refers to the physiological state of the human body. Nevertheless, compounds showing higher concentration in exhaled breath than in indoor air are not exclusively the result of metabolic processes, but can also be stored in different body compartments as a consequence of particular food consumption, smoking, medication, toothpaste, cosmetics and other sources. Some compounds, which could be important as potential biomarkers, are widespread in ambient air especially in a clinical environment, e.g. isopropanol.

Several compounds were *excluded *from consideration (B) due to possible indoor air contamination (p-xylene, isopropanol, N,N-dimethyl formamide), due to their release by Tedlar bags (N,N-dimethyl acetamide, phenol) or GCMS septa (carbon disulfide), due to their appearance in cleaning agents or foodstuff (limonene, p-cymene, menthol) and due to potentially exogenous origin (halogenated compounds), see Table 8. The compound N,N-dimethyl-acetamide is released from Tedlar bags and the compound carbon disulfide (CS_2_) is released by GCMS-septa. Some compounds need a more detailed investigation, such as the ester methyl acetate. This compound might appear in exhaled breath of healthy volunteers at low concentrations (ca. 1 ppb), and has been demonstrated to increase in concentration with increasing cardiac output (in one volunteer, only, results not shown). Other compounds like 2-methyl pentane and 3-methyl pentane are potentially interesting for cancer screening, but might be released from certain types of GCMS-septa, even though not released by the septa used in the present investigation. Ethyl-benzene is a potentially interesting compound but excluded here because it is one of the volatile BTEX-compounds (= benzene, toluene, ethyl-benzene, xylene) appearing in gasoline, which are ubiquitous due to the contamination of soil and groundwater with these compounds.

For distinction between lung cancer patients and healthy volunteers we also excluded (C) all the compounds related to smoking behavior. A typical example is acetonitrile. The concentration of acetonitrile in exhaled breath of smokers is around ~30-60 ppb, whereas the concentration in exhaled breath of non-smokers is low (~2-5 ppb).

Identification based on spectral library match *only *may be misleading (D). We therefore add retention time for proper identification and have built up a database of ~300 physically available compounds (stored in a refrigerator). Since many of the exhaled compounds observed here have never been described in the biochemical literature, it is of utmost importance for further biochemical investigations to confirm the peak attribution with certainty. Therefore identification of peaks based on calibration mixtures (starting from pure compounds) is necessary. Only such a validated identification allows to investigate the biochemical background of the observed compound by, e.g., ^13^C-labelling of metabolic precursors.

We strictly identify only one compound per GCMS-peak (E). Even though this may sound trivial, we know that other researchers attribute more than one compound to a peak (to circumvent the difficulty of chosing one among the several suggestions given by the exclusive use of spectral library identification without checking the retention time).

Collection of exhaled breath has to be done with great care. In particular, all alternative exogenous sources of volatiles should be avoided. We therefore use only Teflon tubings (F) and avoid all other materials, which might release plasticizers.

Altogether 53 compounds were observed in (some) cancer patients but not in healthy volunteers at our sensitivity level of GCMS-SPME. By considering different sets of candidates for cancer marker compounds (set-A: 4 compounds; set-B: 15 compounds; set-C: 21 compounds), we arrived at a specificity of 100% and a sensitivity of 52% (71% and 80%, respectively for the different sets of compounds). Considering all 53 compounds would not increase the sensitivity. Our primary aim was not to achieve high sensitivity for detection of lung cancer, but to identify the compounds observed in exhaled breath of lung cancer patients (of all disease stages) in a careful manner, taking into account not only spectral library match, but also retention time using standards prepared from the respective pure compounds. The mentioned 21 compounds do *not *include smoking-related compounds and do *not *include the compounds given in Table 8, which were *excluded *from consideration. The compounds which we presented here as candidates for cancer marker compounds were not tested in an independent cohort.

SPME-GCMS identifies compounds, but is a relatively *insensitive *method. Hence compounds not appearing in healthy volunteers' exhaled breath may appear at lower concentrations below the limit of detection (LOD). In addition, SPME-GCMS is only semiquantitative due to *competitive absorption *on the SPME-fibre. PTR-MS, on the other hand, does not need preconcentration and gives much more reliable *quantitative *results. The shortcoming of PTR-MS is that it cannot identify compounds with certainty, and several compounds may overlap on a particular mass-to-charge ratio [46]. Hence SPME-GCMS and PTR-MS complement each other, each method having its particular advantages and disadvantages.

In *PTR-MS measurements *we looked at relatively large groups of lung cancer patients (n = 220) and healthy volunteers (*n *= 441). We use tentative identifications for benzene (m/z 79), isoprene (m/z 69), acetone (m/z 59) and methanol (m/z 33). Benzene clearly is a compound related to smoking behavior. The respective concentrations of isoprene, acetone and methanol are lower in lung cancer patients as compared to healthy volunteers. This might partly be an effect of age (as in isoprene [33]), and partly might be due to lower exhalation force in lung cancer patients.

Our healthy controls are younger than patients, and this is particularly so for the GCMS-measurements. Also, no disease controls have been considered, e.g., controls suffering from lung diseases such as COPD. Certainly age, gender and lung diseases (other than lung cancer) are confounding variables, which have to be evaluated very carefully, cf. refs [33,50].

We did not find pronounced differences in composition of exhaled breath between different stages and different treatment regimes. Small differences occur for isoprene, acetone and methanol (PTR-MS measurements). We discussed isoprene in more detail as an illustrative example (see Figures 3, 4 and Table 6): A statistically significant difference in concentration of isoprene was observed between female lung cancer patients and female healthy controls (p < 0.00001). For male lung cancer patients, the respective p-value (p = 0.022) was not below our chosen treshold (namely 0.01) for significance.

The biochemical background of the compounds observed in exhaled breath is largely unknown and needs further elucidation in the future before using certain compounds as biomarkers. In order to be on the right track, it is absolutely compelling to have a proper identification of the compounds observed by GC-MS analysis. It will also be desirable to determine the kinetic rate constants for the exchange of compounds (e.g., isoprene) between different compartments of the body. This is, in particular, important for compounds which are lipophilic, such as isoprene [53,54] or such as methylated hydrocarbons.

The final goal of our investigations is the development of a clinically applicable screening test for detection of lung cancer. Even though our results are promising, there is still a long way to go. We shall need more detailed information about the composition of exhaled breath in cohorts suffering from other carcinomas and other lung diseases, and get information on compounds released from primary cancer cell cultures. Preferably combined information on primary cancer cell cultures and exhaled breath of one and the same patient should be collected.

## Conclusion

Exhaled breath analysis is a promising diagnostic method for detection of lung cancer. Typical compounds arising in everybody's exhaled breath are isoprene, acetone and methanol, which exhibit decreased concentrations in breath of lung cancer patients as compared to healthy controls. Many other compounds arise in lung cancer patients only (when detected by SPME-GCMS with a lower detection limit of ~2-3 ppb). An important issue is the validated identification of volatile compounds observed in breath by comparison with commercially available pure compounds, since many observed compounds from exhaled breath have not been considered before in medical or biochemical context.

## Competing interests

Anton Amann declares to be a representative of Ionimed Analytik GesmbH (Innsbruck). Nevertheless *no *financial reimbursements, fees, funding, or salary have been payed by Ionimed Analytik GesmbH in the last five years. Ionimed Analytik GesmbH was a consortium partner in the EU-project BAMOD.

Anton Amann does *not *hold any shares or patents of an organization which in any way might gain or lose financially from the publication of this manuscript.

The other authors declare that they have no competing interests.

## Authors' contributions

The original plan of the workpackage "lung cancer study" in the EU-project BAMOD was devised and written by JS, WM and AA. GCMS-methodology was developed by JS and WM. The project BAMOD was coordinated by AA.

AB performed patients' interviews and documentation, collection of samples, PTR-MS measurements, wrote parts of the manuscript. CA performed GCMS data analysis and validation. MP performed patients' interviews and documentation, collection of samples, PTR-MS measurements. MK performed patients' interviews and documentation, collection of samples, PTR-MS measurements. KS performed the PTR-MS data analysis, wrote parts of the manuscript. ML performed GCMS measurements of patient samples, indoor air samples and calibration samples, determined the retention times of compounds, did integration of results, wrote part of the manuscript. TL performed GCMS measurements of patient samples, indoor air samples and calibration samples, determined the retention times of compounds, did integration of results, wrote part of the manuscript. WF performed PTR-MS calibration measurements. HD recruited patients, discussed the GCMS and PTR-MS results and compared volatile analyses to patients' medical status and histology of cancer, revised the manuscript. MF recruited patients, discussed the GCMS and PTR-MS results, and compared volatile analyses to patients' medical status and histology of cancer, revised the manuscript. WH recruited patients, discussed the GCMS and PTR-MS results, and compared volatile analyses to patients' medical status and histology of cancer, revised the manuscript. WW recruited patients and discussed the GCMS and PTR-MS results of patients under radiotherapy. PL recruited patients, discussed the GCMS and PTR-MS results of patients under radiotherapy and revised the manuscript. HJ recruited patients and discussed the GCMS and PTR-MS results. MH recruited patients, discussed the GCMS and PTR-MS results, and compared volatile analyses to patients' medical status and histology of cancer. AH assisted in preparing the recruitment protocol and the ethics commission application. BB worked at GCMS protocol planning and measurements, finalized the manuscript. WM planned the original study and the EU-project BAMOD, devised the temperature program and GCMS protocol, provided validation of results and finalized manuscript. JS planned the original study and the EU-project BAMOD, devised the temperature program and GCMS protocol, provided validation of results and finalized manuscript. AA planned the original study and the EU-project BAMOD, supervised all experiments and measurements, validated results, provided software for data analysis and validation, wrote the manuscript. All authors read and approved the manuscript.

## Pre-publication history

The pre-publication history for this paper can be accessed here:

http://www.biomedcentral.com/1471-2407/9/348/prepub
